# The Impact of Artificial Intelligence on the Chess World

**DOI:** 10.2196/24049

**Published:** 2020-12-10

**Authors:** Delia Monica Duca Iliescu

**Affiliations:** 1 Transilvania University of Brasov Brasov Romania

**Keywords:** artificial intelligence, games, chess, AlphaZero, MuZero, cheat detection, coronavirus

## Abstract

This paper focuses on key areas in which artificial intelligence has affected the chess world, including cheat detection methods, which are especially necessary recently, as there has been an unexpected rise in the popularity of online chess. Many major chess events that were to take place in 2020 have been canceled, but the global popularity of chess has in fact grown in recent months due to easier conversion of the game from offline to online formats compared with other games. Still, though a game of chess can be easily played online, there are some concerns about the increased chances of cheating. Artificial intelligence can address these concerns.

## Introduction

All major chess events that were to take place in the second half of 2020 have been canceled, including the 44th Chess Olympiad and the match for the title of World Chess Champion. However, a lot of major national and international chess competitions were recently played online for the first time in history. Through the course of the COVID-19 pandemic, chess has been one of the few sports that has managed to remain relevant to the public and even gain popularity [1], with fans watching online chess tournaments from their homes.

Even though a game of chess can be played online in almost the same way it is played offline, there are some concerns about the increased possibilities of cheating, and many software companies are looking for a solution.

## Context: Artificial Intelligence Progress Displayed in Chess

Artificial intelligence is undergoing a revolution, but at the same time, it has caused revolutionary changes in the world. Chess has inspired artificial intelligence progress for decades [2]. The developments in artificial intelligence for chess have advanced beyond gaming, changing the way machines and humans coexist. The progress of artificial intelligence, highlighted by strategic games, has affected many other areas of interest, as has already been seen in recent years.

One of the most famous human versus machine events was the 1997 victory of Deep Blue, an IBM chess software, against the famous chess champion Garry Kasparov [3]. However, this victory was mainly achieved by brute force, since the Deep Blue program searched millions of positions per second to play chess at a slightly higher strength (the program won the 6-game match with Garry Kasparov with a difference of only 1 point), so it was probably more impressive that Kasparov, using human intuition, could still be almost as strong as a computer searching millions of positions per second. In the following years, computing power advanced to the point where even the best chess players had no chance of defeating a modern chess engine, as has been previously stated [4].

Significant progress in artificial intelligence–related fields was made only 20 years later when AlphaZero won a chess match in 2017 against Stockfish, one of the most powerful chess engines ever created, after a self-learning process of only 4 hours.

The AlphaZero algorithm did not try to use the brute force of computing power to identify as many moves as possible on the chessboard. Instead, using reinforcement learning, humans’ learning process was mimicked by studying an impressive number of chess games [5]. Such interaction with the environment is how people learn. Reinforcement learning, whereby an agent tries to maximize the reward in a “complex, uncertain environment” [6], is just a computational approach to interactive learning. The creators of AlphaZero claimed that the algorithm can learn to optimize decisions in any scenario without changes or guidance [7], and this was truly a breakthrough.

Furthermore, an algorithm that is even beyond AlphaZero was released just a year ago. On November 19, 2019, DeepMind launched the latest algorithm based on reinforcement learning, MuZero. The MuZero algorithm learned to play chess better than AlphaZero without even initially being told the rules of the game. The MuZero learning algorithm has a maximum of 1 million chess games saved in the buffer, with 3000 games played in parallel, as has been stated by Schrittwieser et al [8]. This was possible because DeepMind had access to Google’s vast cloud infrastructure and used thousands of tensor processing unit chips specifically designed for neural network calculations.

There is no way to calculate every possible move on the chessboard up to the end of the game due to the number of possible positions in chess. The first tablebases that tried to solve at least the 5- to 6-piece chess endgames were developed by Ken Thompson [9]. The large amount of storage required to do this makes it impossible to realize this idea for all possible chess positions even to this day.

Thus, the AlphaZero program’s learning of the game of chess in 4 hours with only the rules of the game was remarkable. Still, in the real world, these rules are rarely known. Therefore, the new MuZero algorithm, which manages to play chess on the same level as AlphaZero without receiving these rules, is even more spectacular. Later, even open-source engines such as Leela Chess Zero managed to reach the MuZero level and even surpass it. This was possible using computer power and improvements to the source code provided by a large number of volunteers around the world [10].

## Artificial Intelligence Used for Cheat Detection in Chess Tournaments

It was thought for a long time that people would use artificial intelligence to learn how to play chess better, but now it is successfully used to detect if some contestants play better than they should, considering their game history. Still, these cheat detection mechanisms come with several controversial issues of their own.

As mentioned in the Anti-Cheating Guidelines published by the International Chess Federation (FIDE) in 2014, in most cases, a handheld metal detector is enough to ensure that electronic devices are not being carried into the playing venue, providing cheating protection for onboard games. However, for online chess games, cheat detection has proven to be much more difficult. Almost 4000 players coming from 55 European federations registered for the European Online Chess Championship that took place in May 2020, which was a record number of participants in an official international chess championship. A total of 5 out of 6 players at the top of the B group (rating of 1400-1700) of the European Online Championship were disqualified. In total, more than 80 participants have been disqualified in all categories, which is approximately 2% of the players, the majority being from the beginners or youth categories. Numerous disqualifications at the first-ever European Online Chess Championship highlighted the greatest challenge faced by online chess: cheating [11].

Recently, various software companies, including DeepMind, which developed MuZero, have been working hard to improve existing cheat detection software or even to develop completely new software that will be able to estimate with almost 100% accuracy whether a player is cheating. For example, on Chess.com, a cheat detection system uses millions of chess games stored in its database to create a statistical model that assesses the low probability that a human player will match the top choices of an engine or even surpass the games of some of the greatest chess players in history. All reports of possible cheating are then carefully analyzed by a team of experts. The results are published every month in the website’s “Month in Review.”

Most of the games played during this period have been rapid chess, and chess games played with a longer time per player will be an even bigger problem, as there will be more time for possible forbidden electronic assistance. FIDE has already approved a complex cheat detection technology and an artificial intelligence behavior-tracking module for the FIDE Online Arena games. These online competitions were previously considered separate from the onboard ones, but in the recent pandemic context, the distinction between offline and online has been diminished, with many official competitions being played online for the first time in history.

## Cheat Detection Issues

There are some sensitive issues that should be considered regarding online playing and fraud detection. First, there is a possibility of a noncheating player being classified as a cheater. This would clearly cause serious problems in the career of such a chess player. It is difficult to make this type of analysis for a chess player in their first game ever recorded, but after playing several games in an official online tournament, these programs can easily identify the unlikely differences between normal plays and the plays in which chess engines are used.

Trying to use chess engines every time to play perfectly is not plausible either; this is also easy to detect, as the player would rank first, and in this case, the play can be evaluated by a human expert. Consequently, for those whose playing fluctuates because they sometimes rely on their own decisions and sometimes use chess engines, the new type of cheat detection software based on artificial intelligence detects this very well, with much higher speed and accuracy than a human referee. All suspected cases of fraud are then checked individually by a team of experts in the field before being made public.

The software uses a particular kind of algorithm to detect an unlikely chess move from a specific player. To catch an alleged cheater, the software assesses a set of chess positions played by a certain player (preferably at least a few hundred but the analysis can also work with just a few games) and translates them into a personal performance rating. This helps determine a nonplausible move by using a machine learning algorithm based on clustering, and the result is a list of flags indicating cheating. Of course, when there is a higher number of chess games played by a specific player in the training set, the program will have higher accuracy.

Cheat detection algorithms must be applied carefully to top players because whenever one of them makes a novel move, it could trigger the fraud signal. Still, we can assume that the best players in the world would not risk their reputation over a tournament. It is thought that practicing chess develops certain intrinsic qualities related to fairness, which is probably why most performance chess players never cheat. However, the growing popularity of online chess, especially in recent months, has led to an increase in the number of people completely outside of the chess world trying to win major online tournaments, and some of these people have tried to use chess engines to play better than they normally would at the chessboard.

## Conclusions

What is important for progress in the field of artificial intelligence is that we can measure any breakthrough in machine learning algorithms, for example, through the chess engines that are using them. In other words, it could be argued that chess is a battleground of artificial intelligence, as it is the perfect way to test the battle between human intuition and massive computing power. Due to the complex but well-defined nature of strategic games in general, they have proven to be a perfect environment for testing any progress in artificial intelligence.

Competing against other online players is a bigger part of chess competitions today than ever before, so is not surprising that online fair play is taken more seriously both by chess players who see cheating as a huge impediment to their entire game experience and by companies who create cheat detection software.

Cheat detection software has already been used for important games onboard, even before the COVID-19 pandemic, but this period of forced digitalization has meant that the implementation of cheat detection software has been done on a large scale, even for entertainment games.

We have reached a fundamental question about the entertainment value of chess: What do people really want to see—chess games played between chess players who are sometimes wrong (this being probably the most interesting and educational part to watch) or games played by chess engines, which are beautiful but painfully perfect? Moreover, how do we evaluate the entertainment value or the educational power of a chess game? It is important to analyze what beauty really is in chess. Algorithms for beauty detection in chess diagrams are currently being developed, but this is a topic that needs to be addressed in a future paper.

## Abbreviations

FIDE: International Chess Federation
